# Factors predicting complications following open reduction and internal fixation of intra-articular distal radius fracture

**DOI:** 10.3389/fsurg.2024.1356121

**Published:** 2024-03-22

**Authors:** Lingde Kong, Hua Li, Yanqing Zhou, Bing Zhang, Quan Han, Meng Fu

**Affiliations:** ^1^ Department of Orthopaedic Surgery, The Third Hospital of Hebei Medical University, Shijiazhuang, Hebei, China; ^2^ Department of Hand and Foot Surgery, Hengshui People’s Hospital, Hengshui, Hebei, China; ^3^ Department of Orthopaedic Surgery, Hengshui Sixth People’s Hospital, Hengshui, Hebei, China; ^4^ Medical Examination Center, The Third Hospital of Hebei Medical University, Shijiazhuang, Hebei, China

**Keywords:** intra-articular distal radius fracture, complication, predictors, patient counseling, risk assessment

## Abstract

**Objective:**

This study aimed to determine the incidence and predictors of the complications after open reduction and internal fixation (ORIF) of intra-articular distal radius fracture (IADRF) with a minimum follow-up of 12 months.

**Methods:**

Medical records and outpatient follow-up records were retrospectively reviewed to collect medical, surgical, and complication data on consecutive patients who had undergone an ORIF procedure for an IADRF between January 2019 and June 2022. Data included demographics, comorbidities, injury, surgical characteristics, and laboratory findings on admission. A multivariate logistic regression model was constructed to identify the significant predictors, with a composite of any complications occurring within 12 months after the operation as the outcome variable and potentially a range of clinical data as the independent variables. The magnitude of the relationship was indicated by the odds ratio (OR) and the 95% confidence interval (CI).

**Results:**

During the study period, 474 patients were included, and 64 had documented complications (*n* = 73), representing an accumulated rate of 13.5%. Among them, carpal tunnel syndrome was the most common, followed by tenosynovitis caused by tendon irritation/rupture, superficial or deep wound infection, complex regional pain syndrome (CRPS) type 1, radial shortening (≥4 mm), plate/screw problems, and others. The multivariate results showed the following factors significantly associated with increased risk of complications: experience of DRF surgery with <30 cases (OR: 2.2, 95% CI: 1.6–3.5), AO type C fracture (OR: 1.7, 95% CI: 1.2–2.9), initial lunate facet collapse of ≥5 mm (OR: 4.2, 95% CI: 1.4–8.9), and use of temporary external fixation before index surgery (OR: 2.4, 95% CI: 1.5–4.3).

**Conclusions:**

These findings may aid in patient counseling and quality improvement initiatives, and IADRF should be directed by an experienced surgeon.

## Introduction

Distal radius fractures are frequently encountered in both the emergency and orthopedic departments. Intra-articular distal radius fracture (IADRF) is often caused by high-energy trauma in younger individuals and low-energy falls in osteoporotic elderly patients, with less favorable clinical results (1–4). Surgical intervention through open reduction and internal fixation (ORIF) remains the current standard of care in restoring mechanical alignment, articular congruity, and ligamentous stability, facilitating early mobilization (5). However, substantial postoperative complications compromise the surgical efficacy and functional recovery of the wrist joint. The relevant literature indicated an overall complication rate ranging from 6% to 80% (6), with revision surgery required in 2% to 34% of cases (7–9).

The identification of predictive factors for complications allows for tailoring perioperative care, which has the potential to reduce complications and improve surgical outcomes for patients with IADRF. Multifaceted efforts have been made to address this important issue and identified a broad range of useful or practical predictors, including high-energy trauma, open fracture, greater severity of fracture, involvement of significant lunate fossa collapse, poor bone quality, undesirable placement position of locking screws, comorbid chronic obstructive pulmonary disease, and the lack of experience of surgeons (10–16). Nwosu et al. (17) conducted a systematic review of randomized controlled trials assessing the complications after volar locking plating of distal radius fractures and found a total complication of 30.8%, with major complications accounting for 12.4%. This underscores the importance of identifying potential risk factors, particularly those that are modifiable, from a cost-effectiveness perspective, as most major complications necessitate readmission and secondary surgical interventions (18). In a previous study of the relationship between surgeon experience and the risk of early complications of volar plating of distal radius fractures, the authors suggested that “many of these early complications are avoidable” through centralization of fracture treatment to experienced surgeons (12).

This study aimed to further investigate the incidence and risk factors associated with complications following surgery of IADRF.

## Methods

### Inclusion and exclusion criteria

This study retrospectively searched for the hospitalization register to identify patients who had undergone an IADRF surgery at our institution between January 2019 and June 2022 and further identified those who had at least one complication within 12 months after surgery, by reviewing medical records and follow-up registrations. Prior to the commencement of this study, the protocol was approved by the Ethics Committee of the Third Hospital of Hebei Medical University, which waived the requirement for informed consent of participants because the data were anonymized. This study was conducted in accordance with the Declaration of Helsinki.

The inclusion criteria were adult patients (≥18 years) who had undergone surgery for a fresh isolated IADRF within 14 days after injury and had at least 12 months of follow-up data. The exclusion criteria were patients who had a delay of surgery for >14 days, pathological or metastatic fractures, multiple trauma, concomitant radial or ulnar diaphyseal fractures, previous pathology, operation, or fracture of the affected wrist, patients without reviewable initial radiographs or postoperative radiographs before fracture union was achieved, or patients with incomplete follow-up data.

The surgical indications were an unstable fracture, defined in accordance with the guidelines of the American Academy of Orthopaedic Surgeons (AAOS) (19), as radial shortening ≥3 mm, metaphyseal comminution, dorsal tilt >10°, or intra-articular step-off or displacement ≥2 mm.

### Identification of complications and data collection

Postoperative complications were identified by retrospectively reviewing patient medical records for the index hospitalization and follow-up visit register. These complications included fracture loss or malreduction, plate or/and screw loosening, screw being too long, penetration of screw into articular surface, damage to blood vessel or ligament, extensor/flexor tendon tenosynovitis or rupture, carpal tunnel syndrome, complex regional pain syndrome (CRPS), wound infection, wound dehiscence, fracture union issues (non-union, malunion, or delayed union), secondary traumatic arthritis, refracture, etc. The loss of reduction or malreduction was defined as the presence of dorsal radial tilt exceeding 10°, volar tilt exceeding 20°, or ulnar variance of 3 mm or more, as compared with initial postoperative x-rays (12).

### Variables of interest

The variables of interest were collected and recorded by reviewing patient hospitalization medical records, radiographs, and operative notes, including demographics, smoking status, body mass index (BMI), comorbidities (hypertension, diabetes, heart disease, cerebrovascular disease), fracture mechanism, fracture side, fracture type based on the AO classification, involvement of the lunate facet, time from injury to operation, anesthesia mode, American Society of Anesthesiologists (ASA) grade, timing of operation, surgical emergency, temporary external fixation, surgical approach, surgical duration, intraoperative bleeding, need for blood transfusion, and use of bone graft.

### Statistical analysis

The continuous variables were expressed with mean and standard deviation (SD), and the differences between groups were examined using Student's *t*-test or Mann–Whitney *U*-test, as appropriate, based on their normality status. The categorical variables were expressed with prevalence and percentage, and the differences between groups were examined using the Chi-square test or Fisher's exact test.

The variables that were tested with *p *<* *0.20 were further entered into the multivariate model to evaluate their independent effect on the incidence of complication. In this step, a binary logistic regression model was constructed, using the stepwise backward elimination method. The Hosmer–Lemeshow test was used to evaluate the goodness-of-fit of the final model, with *p *> 0.05 and adjusted Nagelkerke *R*^2^ < 0.750 considered as acceptable results (20). The magnitude of the association with the incidence of complications was indicated by the odds ratio (OR) with a 95% confidential interval (95% CI). *p* < 0.05 was considered statistical significance.

All statistical analyses were performed by SPSS24.0 (IBM Corporation, NY, USA).

## Results

**Figure 1:**
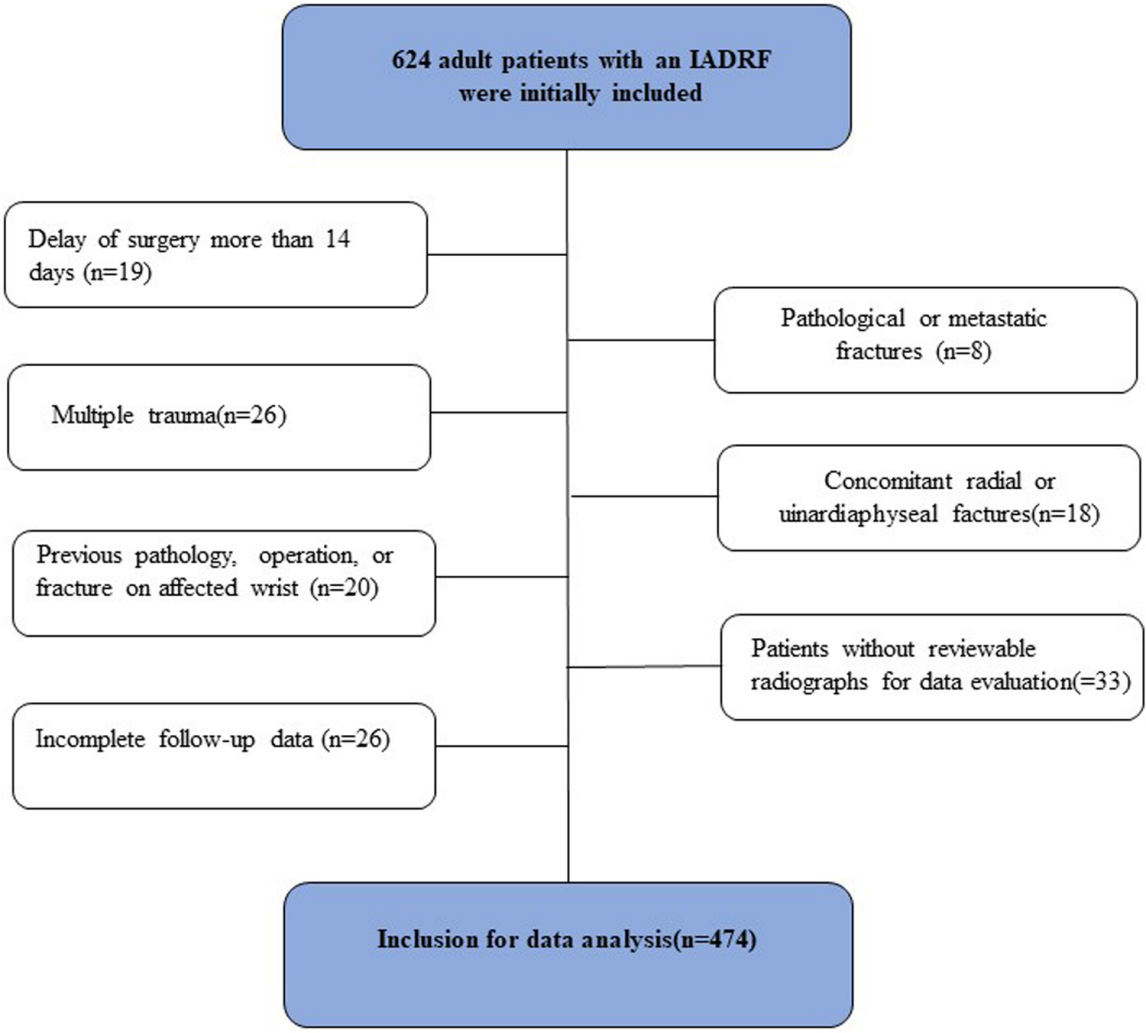
Flow diagram showing the patient inclusion in the study.

In this study, 474 patients, of whom 247 were male and 227 were female, were finally included for data analysis (Figure 1). The mean age was 48.9 ± 14.3 years, ranging from 18 to 85 years, and 87.3% of patients aged <65 years. More than two-thirds (321/474) of the fractures were caused by low-energy trauma, and 54.6% were classified as type C according to the AO classification. The patients were operated on a mean duration of 3.7 days after fracture, and 15.6% had a temporary external fixation to alleviate pain and swelling or stabilize the fracture. In addition, 93.0% of the surgeries were completed during the day and only 7% at nighttime. A total of 17 surgeons, including 10 trauma surgeons and 7 hand surgeons, performed all the procedures, with a median of 14 procedures (interquartile range, IQR: 7–23), and over 90% of the procedures were completed by surgeons who had experience with ≥30 cases before the index procedure.

During the 1-year postoperative evaluation, 64 were found to have 73 complications documented in their medical records or follow-up visit register, representing an accumulated rate of 13.5%. Among them, carpal tunnel syndrome was the most common (14, 21.9%), followed by tenosynovitis caused by tendon irritation/rupture (11, 15.1%), superficial or deep wound infection (9, 12.3%), CRPS type 1 (8, 11.0%), radial shortening (≥4 mm) (8, 11.0%), plate/screw problems (6, 8.2%), and others (Table 1).

**Table 1:** Complications following surgery of IADRFs.

Complication	Number and proportion (%)
Carpal tunnel syndrome	14 (21.9)
Tendon irritation/rupture	11 (15.1)
Superficial or deep wound infection	9 (12.3)
CRPS type 1	8 (11.0)
Radial shortening (≥4 mm)	8 (11.0)
Plate/screw issues	6 (8.2)
Reduction loss	5 (6.8)
Nerve irritation, paresthesia, or numbness	5 (6.8)
Arthritis	4 (5.5)
Delayed union	2 (2.7)
Non-union	1 (1.4)
Total	73 (100)

CRPS, complex regional pain syndrome.

Univariate analyses showed significant differences between the two groups in terms of BMI, injury mechanism, type of fracture according to the AO classification, concomitant lunate facet, use of bone graft, surgeons' experience of DRF management since practice, intraoperative bleeding, and temporary external fixation (Table 2). These variables, together with those with *p* > 0.05 and *p* < 0.20 (time of surgery, surgical emergency, surgical approach), were entered into the multivariate logistic regression model. The multivariate analyses showed that inexperience of DRF surgery (<30 cases) (OR: 2.2, 95% CI: 1.6–3.5), AO type C fracture (OR: 1.7, 95% CI: 1.2–2.9), and initial lunate facet collapse (≥5 mm) (OR: 4.2, 95% CI: 1.4–8.9) and the use of temporary external fixation before index surgery (OR: 2.4, 95% CI: 1.5–4.3) were significantly associated with an increased risk of complications (Table 3). The goodness-of-fit of the final multivariate model was acceptable, with *p* = 0.430 and adjusted Nagelkerke *R*^2^ = 0.392.

**Table 2:** Comparisons between patients with and without documented complications for the data.

Variables	Patients with complications documented (*n* = 64)	Patients without complications documented (*n* = 410)	*p*
Gender (male)	37 (57.8)	210 (51.2)	0.326
Age (years)	47.1 ± 14.0	49.1 ± 14.2	0.292
BMI	25.4 ± 3.2	25.3 ± 3.1	0.977
<24.0	20 (31.3)	157 (38.3)	0.054
24.0–27.9	36 (56.3)	167 (40.7)	
≥28	8 (12.5)	86 (21.0)	
Hypertension	8 (12.5)	72 (17.6)	0.315
Diabetes mellitus	10 (15.6)	64 (15.6)	0.998
Heart disease	5 (7.8)	36 (8.8)	0.798
Cerebrovascular disease	3 (4.7)	23 (5.6)	0.763
Current smoking	17 (26.6)	96 (23.4)	0.583
Hospital stay (days)	13.4 ± 6.9	12.4 ± 6.0	0.209
Injury mechanism			0.008
Low energy	29 (45.3)	292 (71.2)	
High energy	35 (54.7)	118 (28.8)	
Affected side			0.920
Left	34 (53.1)	214 (52.5)	
Right	30 (46.9)	194 (47.5)	
Dominant side			0.532
Yes	29 (45.3)	203 (49.5)	
No	35 (54.7)	207 (50.5)	
Fracture type based on the AO classification			0.030
B	21 (32.8)	194 (47.3)	
C	43 (67.2)	216 (52.7)	
Lunate facet collapse	8 (12.5)	23 (5.6)	0.038
Time to surgery (days)	4.0 ± 2.8	3.6 ± 2.6	0.344
Bone grafting	14 (21.9)	46 (11.2)	0.017
Concomitant carpal tunnel release	3 (4.7)	26 (6.3)	0.608
Timing of surgery			0.179
Day	57 (89.1)	384 (93.7)	
Night	7 (10.9)	26 (6.3)	
Surgical emergency			
Emergent	11 (17.2)	54 (10.6)	0.116
Elective	53 (82.8)	456 (89.4)	
Experience of DRF surgery since practice (<30 cases)	10 (15.6)	32 (7.8)	0.041
ASA score			0.363
I–II	52 (81.3)	351 (85.6)	
III–IV	12 (18.7)	59 (14.4)	
Intraoperative bleeding (ml)	134.7 ± 226.2	106.1 ± 166.0	0.009
Intraoperative blood transfusion	4 (6.3)	35 (5.5)	0.794
Surgical duration (min)	116.4 ± 51.7	119.2 ± 53.7	0.572
Anesthesia (general)	8 (12.5)	47 (11.5)	0.810
Surgical approach			0.121
Volar	52 (81.3)	366 (89.3)	
Dorsal	2 (3.1)	12 (2.9)	
Combined	10 (15.6)	32 (7.8)	
Temporary external fixation	15 (23.4)	57 (13.9)	0.048

BMI, body mass index; ASA, American Society of Anesthesiologists; DRF, distal radius fracturer.

**Table 3:** Multivariate results for the risk factors associated with complications following ORIF of IADRF.

Variables	OR	95% CI		*p*
		Lower limit	Upper limit	
Inexperience of DRF surgery (<30 cases)	2.2	1.6	3.5	0.030
Fracture type (type C vs. B)	1.7	1.2	2.9	0.041
Lunate facet collapse	4.2	1.4	8.9	0.003
Temporary use of external fixator	2.4	1.5	4.3	0.012

OR, odd ratio; CI, confidence interval; DRF, distal radius fracturer; IADRF, intra-articular distal radius fracture; ORIF, open reduction and internal fixation.

## Discussion

Surgical treatment has been the standard of care for IADRF, but the high incidence rate of postoperative complications can compromise the surgical results. Consequently, ongoing efforts continue to identify the risk factors associated with these complications (5, 7, 8, 10–17, 21, 22). In this study, we identified inexperience of DRF surgery (<30 cases), AO type C fracture (vs. type B), the initial collapse of the lunate facet (≥5 mm), and the use of temporary external fixation as significant factors associated with complications after ORIF of IADRF.

Our study recorded an overall complication rate of 15.4%, which is consistent with the findings from previous reports (10, 23), higher than that by Hess et al. who observed an overall complication rate of 9.8% for smokers and 5.6% for non-smokers in a retrospective cohort of 417 DRF patients (24), lower than that (27%) in a study that specified unstable DRFs with a palmar locking plate (25), and lower than that (28.7%) in another study that compared the complications between operative and non-operative DRF patients aged 65 years and above (26). There were several possible explanations. First, the characteristics of the participants varied widely between studies, including age (e.g., some focusing on elderly patients) (23, 26), fracture types (AO type C, A to C, or Colles) (23), or focus on the role of a specific variable (e.g., smoking) (24). Second, most studies did not specifically investigate complications but compared two methods, techniques and fixing devices (e.g., operative vs. non-operative, palmar vs. dorsal surgical approach, plating vs. external fixation) (27–30), as well as varied follow-up periods. Third, the variable definitions of a specific complication have also had a significant impact on the overall complication rate. In 2001, McKay et al. (6) found that overall complication rates vary widely, even from 6% to 80%, with significant differences for patient-reported vs. physician-reported data on complications (rate, 21% vs. 27%).

Temporary use of an external fixator is more likely to reflect the complexity of cases and the subsequent definitive surgical procedure, and it is likely a surrogate of the severity of the fracture. From this point of view, the identification of it as a significant factor is not surprising. A more recent study comparing the one-stage approach (direct osteosynthesis) and the two-stage approach (temporary external fixation as a bridge to definitive osteosynthesis) for the treatment of complex distal radius fracture also obtained a similar finding as ours, that is, the tendency toward more implant removal (34.4% vs. 28.7%), more reoperations needed (4.5% vs. 2.7%), and CRPS (13.1% vs. 7.3%) for the two-stage group (31). However, it is of note that that study did not find significant differences in clinical, functional, and radiographic parameters, suggesting that temporary fixation is a viable alternative.

AO type C fracture and initial lunate facet collapse (≥5 mm) reflected increased fracture severity, therefore necessitating more extensive operative procedures. In addition, they pose greater challenges in fracture reduction and reduction maintenance. In their previous study, Wichlas et al. (27) reported an overall complication rate of 6.3%, with the majority (72.2%, 13/18) occurring in type C fracture, significantly higher than that in types A and B (7.2% vs. 4.8%). A type C fracture as a risk factor was also found in our previous study that studied patients who underwent volar locking plating for distal radius fractures with types A–C (10). Similarly in that study, the initial collapse of the lunate facet of ≥5 mm was identified as a significant risk factor both for overall complications and the need for a secondary procedure (10). Furthermore, in Beck et al.'s (14) study of distal radius fractures AO type B3.3 (volar shearing) treated with the volar plate, they reported that ≥5 mm of initial lunate collapse significantly elevated the risk of failure in patients despite the volar plate being properly positioned. We suggest autogenous bone grafting after tunneling as a treatment option for such a refractory injury type to elevate the collapsed fracture fragments and maintain reduction (32–34).

Due to the nature of intra-articular fractures, surgical techniques to reduce and stabilize IADRFs require a steep learning curve; hence, experience plays an important role in reducing complications. In this study, we found that having experience with 30 cases or less since practice was associated with a 2.2-fold increased risk of complications. This finding supported “practice makes perfect” and was consistent with previous studies. Ward et al. (12) examined the relationship between early complications of volar DRFs and the experience of surgeons and found that the first 30 patients experienced significantly more complications than the later series (rate, 37% vs. 17%, *p* = 0.03). In another study examining surgeon volume in relation to the risk of complication, surgeons who had experienced ≥20 cases of procedures had a 4% rate, compared with 10% of those who had experience with <20 cases (35) This finding supports the centralization of the surgical management of complex distal radius fractures to experienced surgeons to reduce complications as much as possible.

There were several limitations to this study. First, the retrospective study design would have caused imprecise data collection due to the recall bias in comorbidities or complications occurring in the very early period, and some mild complications or complications that resolve in a short timeframe are likely to be underreported. In addition, complications were identified mostly due to a review of medical records, further leading to their underreport. Second, most of the complications collected in this study were from index hospitalization medical records, which were primarily reported by physicians and substantially differed from those reported by patients. From this point of view, these complications are somewhat biased and less representative. We also did not classify them as major or minor because the relevant data were not captured. Third, as with every multivariate analysis, the residual confounding remains due to our inherent limitation in design, including the unknown or unconsidered potential factors that were not adjusted in a multivariate model. Some variables could not be quantified (e.g., the number and frequency of cigarettes smoked, whether diabetes is insulin-dependent, the lasting days of temporary external fixator use), or their severity could not be assessed (e.g., soft tissue damage). Fourth, some patients (26, 5.5%) were lost to follow-up, due to changing of telephone information or relocating to an unknown location outside of the region, which was likely random and did not significantly affect the finding. Fifth, the single-center study design may have affected the extrapolation and generalizability of these findings.

In summary, we found a moderate overall rate of complications after IADRF surgery in a relatively large sample of patients and identified four independent factors predictive of complications, namely, inexperience of DRF surgeries (<30 cases), AO type C fracture, initial collapse of lunate facet (≥5 mm), and the use of temporary external fixation. These data contribute to a more personalized assessment of surgical risks for physicians and aid in improving patient counseling before surgery.

## Data Availability

The original contributions presented in the study are included in the article/Supplementary Material, and further inquiries can be directed to the corresponding author.
